# Evaluation of the Potency of Repurposed Antiretrovirals in HBV Therapy: A Narrative Investigation of the Traditional Medicine Alternatives

**DOI:** 10.3390/ijms26041523

**Published:** 2025-02-11

**Authors:** Samuel Chima Ugbaja, Ata Thabo Mokoena, Aganze Gloire-Aimé Mushebenge, Hezekiel M. Kumalo, Mlungisi Ngcobo, Nceba Gqaleni

**Affiliations:** 1 Discipline of Traditional Medicine, School of Nursing and Public Health, University of KwaZulu-Natal, Durban 4000, South Africa; 2 Africa Health Research Institute, Nelson R. Mandela School of Medicine, University of KwaZulu-Natal, Durban 4000, South Africa; 3 Discipline of Pharmaceutical Sciences, University of KwaZulu-Natal, Durban 4000, South Africa; 4 Faculty of Pharmaceutical Sciences, University of Lubumbashi, Lubumbashi 1825, Democratic Republic of the Congo; 5 Drug Research and Innovation Unit, Discipline of Medical Biochemistry, School of Laboratory Medicine and Medical Science, University of KwaZulu-Natal, Durban 4000, South Africa

**Keywords:** HBV therapy, repurposing, ARV, HIV, hepatocellular carcinoma, traditional medicines, computational drug design

## Abstract

Hepatitis B is one of the killer communicable diseases, with a global estimation of 1.1 million deaths resulting from liver diseases annually. The search for HBV therapeutics has resulted in repurposing the existing antiretrovirals (ARVs) for HBV treatment, considering their shared common replication mechanisms. This review is aimed at evaluating the potencies of some of the repurposed ARVs used for HBV treatment, analyzing the common mechanisms of viral replications in HBV and HIV, and investigating the potentials of traditional medicines as an alternative treatment for HBV patients. The topical keywords drug repurposing, drug repositioning, antiretrovirals, hepatitis B treatment, HBV, natural products, traditional medicines, title, and abstract were searched in PubMed, Web of Science, and Google Scholar. The advanced search included the five years, 2019–2024. The search result was filtered from 377 to 110 relevant articles. The evaluation reveals that CD4+ T cells are targeted by HIV, while HBV targets the liver with its associated diseases (cirrhosis and hepatocellular carcinoma (HCC)). Furthermore, treatments with the available repurposed ARVs only prevent or slow down the progression to cirrhosis, reduce the HCC incidence, and can improve the quality of life and increase life expectancy; however, they are not curative for HBV. Traditional medicines/natural product extracts or their phytochemicals exert anti-HBV effects through different mechanisms. Traditional medicines exert improved therapeutic effects when combined properly. The investigation further reveals that consideration of an in silico approach in HBV therapeutics might not only streamline drug development but also contribute to a deeper understanding of viral pathogenesis. Therefore, we recommend the integration of computational drug design methods with traditional medicine and natural product screening for discovering new bioactive HBV drug candidates

## 1. Introduction

**Figure 1:**
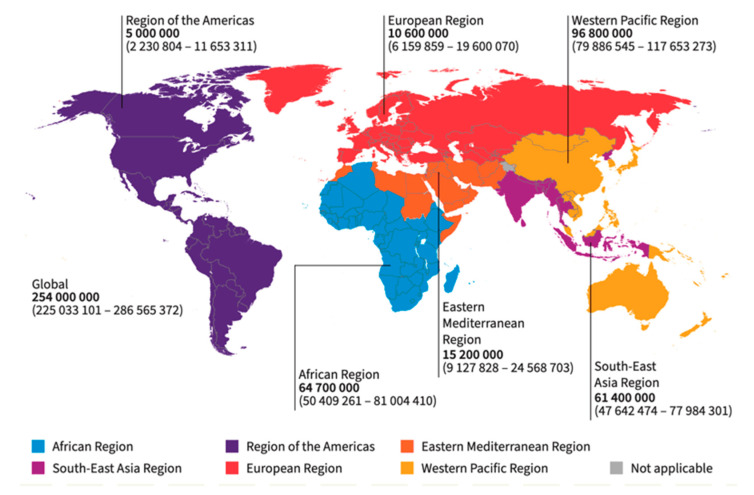
Worldwide distribution of chronic hepatitis B by WHO region as adapted from the source [2,3].

Hepatitis is a major public health concern that causes inflammation of the liver due to hepatitis B virus (HBV) infection [1]. According to the World Health Organization (WHO) 2024 Global Hepatitis Report, the global prevalence of people living with HBV infection is estimated at 254 million, with an estimated incidence of 1.2 million new HBV infections per annum, as depicted in Figure 1 below. Hepatitis B is one of the killer communicable diseases, with an estimated 1.1 million deaths per year caused by cirrhosis and hepatocellular carcinoma (HCC) [2,3]. A study by Veracruz N. et al. (2022) reported a decline in the overall global impact of HBV on disability-adjusted life years (DALYs) per 100,000. The reported 9 years (2010 to 2019) observed a decrease from 27.6 to 20.9 for acute HBV and 168.6 to 129.8 for HBV-related cirrhosis. However, the figures for HBV-related HCC remained unchanged. The authors reported relative global achievement in the fight against HBV, with some observed inequitable access to HBV treatment in some regions [4].

**Figure 2:**
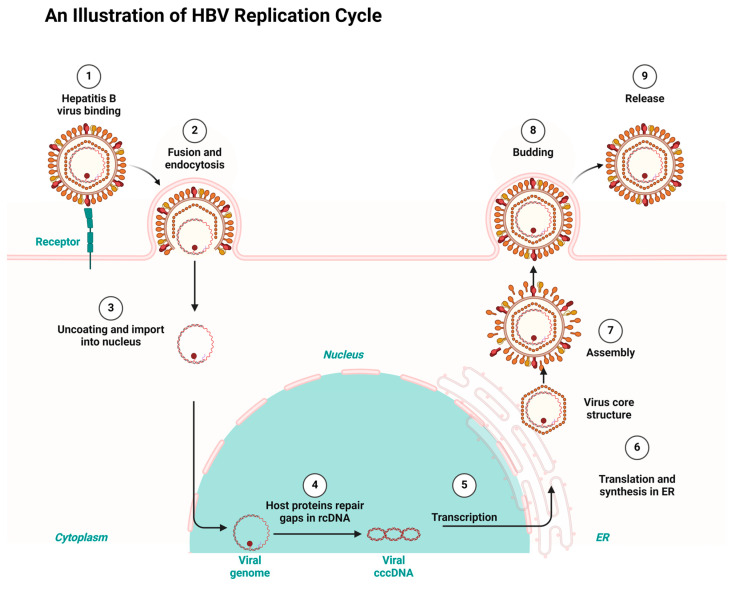
HBV replication cycle highlighting key stages targeted by antiretrovirals for HBV treatment, redrawn with BioRender as adapted from sources [7,8].

It is projected that by 2040, deaths from chronic viral hepatitis, especially from HCC, will double, and this will exceed the combined mortality rates of tuberculosis, human immunodeficiency virus (HIV) infection, and malaria [5,6]. HBV attaches to the host’s cell surface using heparan sulfate proteoglycans. Further binding to sodium taurocholate co-transporting polypeptide (NTCP) facilitates the viral entry, followed by fusion and uncoating. The process enables HBV to transfer its relaxed circular DNA (rcDNA) into the nucleus for replication. Openings in the relaxed circular DNA (rcDNA) are repaired by the host’s proteins and converted into covalently closed circular DNA (cccDNA) in the template for transcriptions. Thereafter, the transcribed RNA is transported to the cytoplasm for synthesis and translation into viral proteins at the endoplasmic reticulum (ER). The accumulation of the viral core structure around the new viral genome leads to the maturation of new infectious viruses, which are discharged to infect new cells. Figure 2 below depicts the HBV replication cycle [7,8].

HBV infections vary according to different geographic areas globally. The Western Pacific and African regions are regarded as highly endemic, accounting for 97 million and 65 million people who are chronically infected with HBV. The African region is estimated to account for 63% of new HBV infections with the majority of these cases acquired during childhood. In addition, two-thirds of the global HBV infections are collectively shared by Bangladesh, China, Ethiopia, India, Indonesia, Nigeria, Pakistan, the Philippines, the Russian Federation, and Vietnam. However, approximately 47% of deaths from HBV occur in the Western Pacific region, 25% in the African region, and 20% in the South-East Asia region [2,9]. In addition, cultural beliefs and wrong assumptions about HBV sometimes result in a lack of interest in modern healthcare systems. Moreover, the preference for indigenous traditional herbal healing practices over approved HBV therapeutics slows the diagnosis of the disease and subsequent treatment. Furthermore, cultural stigmatization of people suspected to be living with HBV demotivates people from participating in voluntary public screening and treatment. As previously mentioned, inequality in accessing healthcare and lack of trust in modern medical facilities worsen the prevalence of HBV in marginalized regions. These biases and inequalities could be addressed through community and stakeholder engagement, grassroots sensitization, education, and awareness creation on the benefits of modern healthcare systems [10].

HBV is a non-cytopathic DNA virus that belongs to the Hepanaviridae family and can be found in the blood and other bodily fluids, such as saliva, vaginal fluids, and semen. HBV can remain stable on surfaces at 37 °C with a half-life of more than 22 days and can be transmitted via vertical or horizontal transmission, and parenteral contact with infected blood or blood products [11,12,13]. In areas of high endemicity, especially some regions within Asia and the Western Pacific, vertical transmission, which is mother-to-child, is the most prevalent mode of transmission; whereas in sub-Saharan Africa, the Mediterranean region, and Alaska, transmission is mostly via person-to-person contact during preschool years [14,15,16]. In regions of intermediate to low endemicity, such as Western Europe, HBV can be acquired during adulthood through horizontal transmission, which involves unprotected vaginal, oral, or anal sexual intercourse and contact with the infected patient’s mucosal secretions, such as saliva, vaginal secretions, semen, or blood. In addition, HBV can be transmitted using unsafe injections of drugs or exposure to sharp objects [14,16,17]. Exposure to HBV can result in acute hepatitis B (AHB) or chronic hepatitis B (CHB). For AHB, the infection usually happens in adulthood and the immunocompetent individual can eliminate the virus particles. Consequently, CHB results from infection during infancy or in immunocompromised individuals [11]. According to Vega S. et al. (2013), Zinc ion plays an essential role in the stabilization of some proteins, which are crucial for the mechanistic processes of viral polyproteins in infected hepatic cells. For example, protease shows an induced-fit mechanism when complexed with zinc, resulting in a significant change in conformation that improves catalytic efficiency. Therefore, the presence of zinc ions in the biological system is suggested to be essential in the onset and growth of hepatitis virus diseases and influences viral replications and pathogenesis. Controlling such biophysical cues could be a potential strategy for reducing viral replications and fostering the design of targeted antiviral treatment [18]. The WHO Global Health Sector Strategy proposed the elimination of HBV as one of its goals for 2030 and defines it as a 90% reduction in incidence and a 65% reduction in mortality using the 2015 statistics as a baseline. To achieve these goals, the WHO proposed strategies for the elimination of chronic hepatitis, including vaccination, scaled-up testing, improved medical treatment, and public health interventions [19].

Substantial progress has been made in the use of prophylactic vaccines as preventative measures and has been implemented in many countries to control HBV infections. The major global incidence of HBV is mostly attributed to early childhood or mother-to-child infection and the WHO recommended the universal birth-dose monovalent HBV vaccination within 12 to 24 h after the birth of the baby [6]. In addition, large-scale, effective, cheap, and safe HBV vaccines, including Engerix-B (SmithKline Biologicals, Rixensart, Belgium), RECOMBIVAX HB and HB-Vax II (Merck & Co., Rahway, NJ, USA), and TGP 943 (Takeda Chem, Tokyo, Japan) have been administered in medium to high endemic regions. This strategy has resulted in a reduction in childhood HCC and HBV carriage rates [20]. However, the efficacy of Engerix-B is reduced in individuals who are immunosuppressed, HIV-infected, tobacco smokers, have a genetic predisposition, and have other chronic illnesses [21]. It is to be noted that the complete vaccination course requires three doses within a period of six months with the currently available vaccines. Therefore, this can lead to reduced compliance, costs of the vaccine doses, and travel expenses to the healthcare center. However, in 2017, the FDA approved HEPLISAV-B (HepBCpG) as a new, cost-effective, immunostimulatory sequence adjuvant HBV vaccine after more than 25 years [22]. This vaccine only requires two dose regimens and had a higher seroprotection in poor vaccine responses, including adults, diabetics individuals with chronic liver disease, and chronic kidney disease. However, these vaccines are only effective in people who have not been infected with HBV, and chronically infected patients must rely on immunomodulatory agents or repurposed antiviral drugs [23,24,25,26,27].

Several therapeutic options for HBV infections have been approved by the U.S. Food and Drug Administration (FDA), which include nucleoside analogs such as entecavir (ETV), lamivudine (LAM), and telbivudine (LdT). Nucleotide analogs include adefovir dipivoxil (ADV), tenofovir disoproxil fumarate (TDF), and tenofovir alafenamide (TAF). Interferons include peginterferon α-2β/2α and the addition of polyethylene glycol (PEG) [6,28,29,30]. The use of these available treatments can prevent or slow down the progression to cirrhosis, reduce the HCC incidence, improve the quality of life, and increase life expectancy. However, they are not curative for HBV [24]. Therefore, patients using these medicines are required to stay on indefinite treatment; thus, patient management and monitoring can become complex. Interferons play a huge role as antiviral and immunoregulatory agents to treat HBV; however, they have limited efficacy and can cause adverse side effects, including bone marrow suppression, aggravated neuropsychiatric symptoms such as depression, flu-like symptoms, thrombocytopenia, leukopenia and anemia [31,32,33]. Nucleosides or nucleotides have been shown to suppress HBV replication and, thus, reduce the viral load; however, they can have tremendous side effects, including renal toxicity, mitochondrial toxicity, effects on bone density mineral density, myopathy, and drug resistance [34,35,36]. According to the WHO 2024 report, approximately 3% (seven million) of people with CHB have been treated and 13% diagnosed with HBV. Therefore, these results are still not close to the global target of people living with CHB by 2030, but they represent a huge improvement from the previously reported estimates [2]. The curiosity for an HBV cure culminated in the repurposing of existing antiretrovirals for HBV treatment, considering their shared common replication mechanisms.

Even though the repurposing and repositioning of drugs vary slightly, public health researchers use them interchangeably as the same concept. Drug repurposing entails using Food and Drug Administration (FDA)-approved drugs for diseases for which they were not originally approved. Subsequently, drug repositioning involves using an approved drug for a different disease within a similar therapeutic area. The repurposed or repositioned drugs are further subjected to clinical trials before subsequent final approval. The COVID-19 pandemic necessitated the repurposing and repositioning of some drug agents, including tocilizumab, baricitinib, and remdesivir, originally FDA-approved to treat other diseases for the treatment of COVID-19 patients [37,38,39]. Consequently, repositioning already-approved drugs for treating other related diseases is economical because it saves resources (time, processes, and money) that would have been expended on designing new drugs [40]. Recently, antiretroviral (ARV) drugs originally approved for HIV treatment are being used for HBV therapy because both HIV and HBV replicate through a similar pathway called ‘reverse transcription.’ A holistic insight into the mechanistic inhibition of viral replication in HBV patients is key to for effective and potent therapy. Table 1 depicts some examples of ARVs repurposed for HBV therapy, two-dimensional (2D) structures, the mechanism of action, and likely limitations.

Notably, HIV and HBV remain a global burning public health constraint, with millions of newer infections emerging annually. Recently, repurposed ARV drugs have exhibited relative therapeutic potency for HBV treatment. The repurposing of ARVs for HBV treatment is possible because of the similar mechanisms shared by HIV and HBV in viral replication cycles, although they differ in their targets. They both replicate through reverse transcription. While HIV targets CD4+ T cells, the liver with its associated diseases (cirrhosis and hepatocellular carcinoma) is targeted by HBV. Also, both viruses use receptor-mediated entrance, the first shared vulnerability. Table 2 depicts the comparative analysis of HIV and HBV replication mechanisms. An understanding of these mechanisms is essential for drug repurposing.

Following the adverse side effects of some of the repurposed ARVs for HBV treatment, other options, such as computer-aided drug design, otherwise referred to as in silico methods, and traditional medicines, have also recently been explored.

## 2. Therapeutic Potency of Traditional Medicines as Antiviral Agents

Natural products (NPs) can be derived from plants, animals, microorganisms, and fungi as important sources of new drug discovery and alternative therapeutic agents. Plants consist of phytoconstituents that have pharmacological properties, such as antiviral, anti-inflammatory, anti-carcinogenic, antioxidative potentials, and antidiabetic [75,76]. Historically, NPs have been used as traditional medicines (TMs) to treat various human diseases, including infectious, neurological, oncological, cardiovascular, and metabolic [77]. NPs from medicinal plants have complex structures, and their secondary metabolites, such as terpenes, alkaloids, glycosides, and phenolic compounds, are considered better candidates for drug development compared to purely synthetic sources. It has been reported that about 2,140,000 secondary metabolites are found in the plant kingdom, and they have been used to develop some of the currently available medicines, including morphine, taxol, artemisinin, paclitaxel, and calanolide A [78,79,80,81]. In 2023, the FDA Center for Drug Evaluation and Research (CDER) in conjunction with the FDA Center for Biologics Evaluation and Research (CBER) approved 55 new drugs that are known as “novel” drugs as compared to 37 that were approved in 2022. According to the report, 17 were approved as biologics, which are drugs produced from a variety of natural sources, and this accounted for a total of 30% of all drugs produced; whereas this figure was approximately 28% in the last five years (69 biologics from a total of 247 drugs produced) [81,82,83]. In addition, 22 new Biologics License Application approvals were added by the CBER in 2023 compared to 12 added in 2022 [82]. TMs, which can be a single, or multiple combination of medicinal plants, have been shown to have therapeutic effects on various viral infections, including HIV, herpes simplex virus (HSV), severe acute respiratory syndrome coronavirus 2 (SARS-CoV-2), coxsackievirus, influenza, and hepatitis in both clinical and preclinical studies. Their biological activities are screened using various in vitro and in vivo assays based on reported ethnopharmacological and historical uses [84,85].

## 3. Historic Overview of Traditional Medicine Applications in HBV Treatment

The origins of the use of TMs are not clear because it was passed down from generation to generation as oral knowledge through frequent storytelling or spiritually from the ancestors. However, it is believed that the use of TMs was dominant in West Asia, East Asia, Africa, and South and Central America [86]. The history of plant species recorded in Mesopotamia includes Papaver somniferum L., Commiphora acuminata Mattick, and Glycyrrhiza glabra L., which have been used as medicines since 2600 BC, with approximately 1000 plant-based formulae that are in use today to treat various ailments. China is regarded as one of the most dominant countries in the use of TMs and according to the Chinese Materia Medica, traditional Chinese medicine dates to 1100 BCE with over 11,000 plant formulae [87]. In the African continent, the ancient Pharaonic Egyptian medical practices, which date back to 3300 BC, included simple non-invasive surgery, bone setting, and pharmacopeia, and are known as the oldest recorded documents describing the use of TM in Africa. In addition, the Ebers Papyrus, dating back to 1500 BC, contains over 700 remedies and records of diabetes diagnosis as “urine pass through”, while the Edwin Smith Papyrus, dating back to 1600 BC, includes breast cancer and its management as “tumor do thou nothing there against”. These are among the oldest preserved medical documents in the African continent [88].

Scientific studies using ethnopharmacological plants that were in use during ancient times have made substantial progress through in vitro studies to explore crude extracts as inhibitors of the HBV entry, replication, and maturation of particles, and anti-liver cancer properties during the last decade (Table 3). Ethanolic extracts of Scutellaria barbata D. Don (SB) and Oldenlandia diffusa (Willd.) Roxb (OD) inhibited the levels of Hepatitis B surface antigen (HBsAg) and HBV-DNA by affecting the different expressions of transcriptomic RNAs, including circRNAs and miRNAs. In addition to the in vitro replication inhibition studies, SB and OD were also shown to inhibit cellular proliferation of HBV-related HCC cell lines, reduce the number and size of colonies after 14 days of cell culture, and reduce the tumor volume and weight of mice treated with SB, OD, and SB + OD extracts compared to the tumor-bearing control mice [89]. Crude extracts of Cassia fistula and Melastoma malabathricum, dissolved in dimethyl sulfoxide (DMSO), along with the methanolic extract from Cananga odorata, suppressed the production of extracellular HBV DNA and viral entry into the cells, with Cananga odorata being the most effective extract [6].

Furthermore, a study titled “In vitro evaluation of novel antiviral activities of 60 medicinal plants extracts against hepatitis B virus” by Arbab et al., (2017), conducted in Saudi Arabia, investigated the potency of TM ethanolic and organic extracts used for liver disease [91]. The results showed that 9 of the 60 medicinal plants, including Guiera senegalensis, Pulicaria crispa, and Fumaria parviflora, displayed important anti-HBV potential through the inhibition of the two important antigens linked to the HBV (HBsAg and HBeAg) in HepG2.2.15 cells. Additional phytochemical screening showed that secondary metabolites such as alkaloids, tannins, flavonoids, and saponins contributed to the observed antiviral properties. However, further phytochemical analysis was suggested for the isolation of the active compounds responsible for these antiviral potentials [91]. Another study titled “Effects of Ixeris Chinensis (Thunb.) Nakai boiling water extract on hepatitis B viral activity and hepatocellular carcinoma” by Shih et al., (2014) demonstrated the ability of this medicinal plant to downregulate the expression of HBsAg, inhibition of HCC growth, and induction of apoptosis. However, further experiments, including animal models and clinical trials, were recommended to further develop these crude extracts into effective anti-HBV therapeutic agents [92].

Most of the studies conducted to find new anti-HBV therapeutics or HBV-induced HCC were in vitro due to the low cost of these studies. Nonetheless, these crude extracts have minimal reports on their active compounds, toxicity, and safety profiles. Therefore, it would be ideal to further explore studies on their toxicity, efficacy, and pharmacokinetics. The current treatment of HBV infection involves the use of HIV antivirals. The use of medicinal plants in conjunction with ARVs might lead to drug interactions, and toxicity, resulting in treatment failure if not carefully monitored. Furthermore, crude extracts consist of various active compounds, which play a role in synergistic beneficial effects; therefore, it is imperative to further characterize these active compounds in the extracts to develop HBV chemosystematics markers. Crude extracts that have the potential to induce apoptosis are of immense importance as this helps kill virally infected cells and tumor cells [92].

## 4. Enhanced Mechanistic Synergy of Natural Products/Traditional Medicines as Antiviral Agents

Numerous phytochemicals, including flavonoids, phenols, alkaloids, saponins, and terpenoids, among others, have been extensively studied for their liver-protective benefits. Flavonoids are responsible for the plant pigment and are found in fruits, flowers, seeds, and roots of medicinal or aromatic plants, and they have pharmacological benefits, including anti-inflammatory, anti-tumor, anti-oxidant, detoxifying properties, and metastasis effects [93]. Silibinin is a major polyphenolic compound in silymarin, extracted from the seeds of herb milk thistle. A study using two silibinin derivatives with improved solubility as compared to the parent compound demonstrated that these derivatives reduced liver injury, had milder hepatocyte necrosis, reduced levels of serum aminotransferases [glutamate aminotransferase (ALT) and aspartate aminotransferase (AST)] and lower expression of pro-inflammatory cytokines, including TNF-α, IL-6, and IL-1β using CCl4-induced acute liver injury in mice [94]. Subsequently, studies have shown that silibinin reduces liver fibrosis in nonalcoholic fatty liver disease patients and decreases ALT and AST levels in mice using sibilin capsules. The use of silibinin capsules in patients suffering from metabolic-associated fatty liver disease, together with a traditional Chinese medicine Jiangzhi Paizhuo decoction, results in a reduction in the control attenuation parameter measurement, which is a measure of hepatic steatosis in patients with chronic hepatitis B [95,96,97]. Alanine Aminotransferase enzyme (serum ALT) and aspartate aminotransferase enzyme (serum AST) are found in some tissues, such as the liver, and at elevated states indicate damage to the liver or such tissues [98]. Another study titled “Complementary Efficacy of Antrodia cinnamomea Mycelia on Patients with Chronic Hepatitis C Virus Infection: A Randomized Controlled Pilot Clinical Study” by Chung-Hung et al. (2017) demonstrated a decrease in levels of serum AST and ALT in patients suffering from chronic hepatitis C virus infection when using oral supplementation of Antrodia cinnamomea mycelium with pegRiba therapy [99].

The above studies showed enhanced mechanistic synergy of the combined traditional medicines. However, it has been reported that some of the phytocompounds, including aristolochic acid, alkenylbenzenes, and pyrrolizidine alkaloids, have hepatocarcinogenic effects, whereas others can induce nephrotoxicity and cardiotoxicity [100]. HIV antiretrovirals are commonly used as standard therapeutic agents for the treatment of HBV infection but some patients experience side effects or resistance to the ARVs. There are numerous studies focusing on active compounds from medicinal plants that have anti-HIV properties to inhibit viral replication, binding of the virus to the host cells, disruption of the virus life cycle, and immune regulation. Thus, it is important to find new anti-HBV therapeutics or construct new combination regimens that have low toxicity and drug resistance. Clinical studies have demonstrated the use of traditional medicines combined with HIV ARVs to improve treatment outcomes, reduce the hepatoxicity of the ARV, and enhance ARV efficacy through synergistic mechanisms. A study investigating the protective effects of Piper nigrum stem (PNS) against tenofovir/lamivudine/efavirenz (TLE)-induced hepatotoxicity and dyslipidemia in Wistar rats demonstrated that the PNS-treated group had decreased levels of alkaline phosphatase and triglycerides as compared to TLE group [96,97]. The authors observed increased levels of triglycerides in the TLE group, which might lead to the development of dyslipidemia. In addition, an increase in catalase and reduction in glutathione in the TLE group, compared to the PNS group, was also observed, which could lead to liver toxicity in rats due to oxidative stress. Furthermore, the TLE group had leukocyte infiltration, which could induce renal damage [101]. Another study investigating the replication effects of Phyllanthus urinaria L. extract on lamivudine-resistant hepatitis B virus in vitro demonstrated that intracellular synthesis of HBV DNA and HBcAg secretion was inhibited in replicating lamivudine-resistant mutant cells, and the expression of IL-6, IFN-β, and cyclooxygenase-2 (COX-2) mRNA was induced [102].

Lastly, traditional medicines/natural extracts, or their phytochemicals can exert anti-HBV effects through different mechanisms, such as the expression of host and HBV factors, including immunological regulation (inducing IFN-α and β, IL-6, and TNF-α expression), regulating the expression of different HBV proteins, promoting autophagy, and suppressing important pathways in the HBV life cycle, such as polymerase replication and MAPK pathways. Some of these mechanisms differ from the ones employed by the currently available ARVs used for HBV infection treatment. Therefore, in clinical settings, these extracts, together with the ARVs, can exert combined effects through synergistic viral suppression, reduction in hepatocytotoxicity, improved immune modulation and oxidative stress, and alleviation of other side effects caused by conventional anti-HBV drugs [103,104,105].

## 5. Limitations of Traditional Medicines

Despite the promising therapeutic potential of traditional medicines (TMs), there are limitations in the use of these medicines in research studies. The use of TMs is solely based on the traditional health practitioners’ indigenous knowledge of diseases, and these TMs can have toxic effects due to the lack of international health standards evaluations, including the correct dosage and quality controls. Consequently, there is still a lack of well-designed clinical trials to validate the effectiveness and safety of most TMs in human subjects for various ailments, as most studies are in vitro or performed on animal models, and numerous natural products are classified as dietary supplements. However, creating the placebo control product during clinical trials might be challenging because some medicinal plants have a peculiar odor, color, and taste. In addition, there are limited research studies on the mode of action, long-term effects, side effects, and contraindications on the use of TMs. Research using oral preparations of Xianling Gubao has shown that the use of this formulated herb mixture can cause herb-induced liver injury in human subjects and mice [106,107].

Furthermore, there is a lack of standardized extraction methods, compositions, and purity of the extracts by pharmaceutical industries, High-Performance Liquid Chromatography (HPLC) analysis, and authentication of the herbs to ensure consistency and reliability of the results in the different research studies. Traditional medicines can be formulated or produced from a single or mixture of medicinal plants that contain numerous active compounds. The isolation of these requires tremendous time and resources, which might be impossible in a product with more than one medicinal plant. Another challenge is the lack of proper quality control standards, including harvesting, good manufacturing practices, and proper identification and authentication of medicinal plants. Moreover, there is a lack of knowledge and documentation regarding botanical nomenclature to provide the correct scientific name of the plant, compared to the generally used common names. This can lead to misidentification or mislabeling of the medicinal plants and result in the wrong product purchased by the consumer. Therefore, there is a need to explore other alternative HBV drug design methods, such as in silico or Computer-Aided Drug Design methods, and genetic profiling. Park J. et al. (2025) reported the importance of other genomic techniques, such as genome-wide association studies (GWAS), whole-exome sequencing (WES), and whole-genome sequencing (WGS), in detecting uncommon genomic variants responsible for viral replication in individuals living with HBV. Moreso, epigenetic profiling, including deoxyribonucleic acid (DNA)-methylation and histone modifications study can unveil the dynamic mechanisms responsible for HBV pathogenesis. The authors further reveal that single-cell sequencing helps in identifying cellular heterogeneities in the liver. In addition, other genomic techniques, such as Clustered Regularly Interspaced Short Palindromic Repeats (CRISPR)-based screens, could be useful in validating genes such as RGL1, CDCA7L, and AQP9 and their functions in viral reactivation [108].

## 6. Unveiling the Importance of in Silico Methods in HBV Drug Repurposing

The development of novel HBV inhibitors requires innovative strategies to overcome limitations in current methodologies [109]. Computational approaches, including molecular docking, pharmacophore modeling, and molecular dynamic simulations, have proven instrumental in this endeavor. These tools allow researchers to virtually screen millions of compounds and prioritize those with favorable antiviral properties, significantly expediting the drug discovery process. The efficiency of computational methods is evident from their ability to address challenges faced in antiviral research for other viruses like the Hepatitis C Virus (HCV). HCV drug development has benefited from structure-based drug design and virtual screening to overcome issues like drug resistance and lack of specificity [110,111]. These methods provide insights into the mechanisms of action, uncover previously neglected viral targets, and enable the design of selective inhibitors [112].

Similarly, for HBV, in silico approaches can facilitate the identification of novel HBV therapeutic agents by predicting drug–target interactions and optimizing lead compounds [113]. These techniques offer a cost-effective and time-efficient platform to explore and refine drug candidates, addressing the urgent need for effective HBV treatments. Moreover, the COVID-19 pandemic has highlighted the value of repurposing existing drugs using computational strategies. Molecular docking and pharmacogenetic assessments have accelerated the identification of potential therapies by leveraging known pharmacokinetic and pharmacodynamic properties [114]. Applying these lessons to HBV therapy can enhance precision medicine by identifying synergistic combinations and minimizing adverse interactions. Furthermore, integrating computational methods with traditional medicine and natural product screening opens new avenues for discovering bioactive compounds. By bridging gaps in experimental research, in silico approaches not only streamline drug development but also contribute to a deeper understanding of viral pathogenesis and therapeutic intervention.

## 7. Conclusions

HIV and HBV remain a global burning public health constraint, with millions of newer infections emerging annually. Recently, repurposed ARV drugs have exhibited relative therapeutic potency for HBV treatment. The search for HBV therapeutics has resulted in the repurposing of existing antiretrovirals (ARVs) for HBV treatment, considering their shared common replication mechanisms. Antiretroviral (ARV) drugs approved for HIV treatment are being repurposed for HBV treatment due to their common replication pathway, called ‘reverse transcription. This study evaluated the potential of some of the repurposed ARVs used for HBV treatment, analyzed the common mechanisms of viral replication in HBV and HIV, and investigated the potential of traditional medicines as alternative treatments for HBV patients. The repurposing of ARVs for HBV treatment was possible because of the similar mechanisms shared by HIV and HBV in viral replication cycles, although they differ in their targets. They both replicate through reverse transcription. While HIV targets CD4+ T cells, the liver, along with its associated diseases (cirrhosis and hepatocellular carcinoma), is targeted by HBV. Also, both viruses use receptor-mediated entrance, the first shared vulnerability. Subsequently, studies have shown that silibinin reduces liver fibrosis in nonalcoholic fatty liver disease patients and decreases ALT and AST levels in mice using silibinin capsules. The usage of silibinin capsules in patients suffering from metabolic-associated fatty liver disease, together with a traditional Chinese medicine Jiangzhi Paizhuo decoction, resulted in a reduction in the control attenuation parameter measurement, which is a measure of hepatic steatosis in patients with chronic hepatitis B. Furthermore, an enhanced mechanistic synergy of the combined traditional medicines is preferred. Lastly, traditional medicines/natural extracts, or their phytochemicals can exert anti-HBV effects through different mechanisms, such as the expression of host and HBV factors, including immunological regulation (inducing IFN-α and β, IL-6, and TNF-α expression), regulating the expression of different HBV proteins, promoting autophagy, and suppressing important pathways in the HBV life cycle, such as polymerase replication and MAPK pathways. Some of these mechanisms differ from the ones employed by the currently available ARVs used for HBV infection treatment. Therefore, in clinical settings, these extracts, together with the ARVs, can exert combined effects through synergistic viral suppression, reduction in hepatocyte toxicity, improved immune modulation and oxidative stress, and alleviation of other side effects caused by conventional anti-HBV drugs. We, therefore, suggest further integration of computational methods with traditional medicine and natural product screening, which might open new avenues for discovering HBV bioactive compounds. Moreover, considering the importance of genetic profiling in unveiling the dynamic mechanisms responsible for HBV pathogenesis, we strongly recommend its combination with in vivo, in vitro, and other in silico techniques for more efficient and future HBV therapeutics.

## Figures and Tables

**Table 1:** Common ARV drugs repurposed for HBV therapy.

Drug Name	2D Structure	Mode of Action	Limitations	References
Abacavir (ABC)	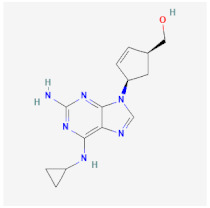	Mostly used for HIV treatment but has limited inhibition of HBV replication due to its inhibition of the reverse transcriptase mode of action.	Exhibits cross-resistance, reduced efficiency, and hypersensitive reaction in HBV treatment.	[41,42]
Lamivudine (3TC)	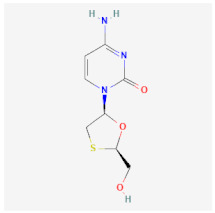	Terminates viral replication through the inhibition of HBV DNA polymerase.	It requires combined therapy and is only good for short-term use due to increased resistance rate at a prolonged usage in monotherapy.	[43,44]
Adefovir Dipivoxil (ADV)	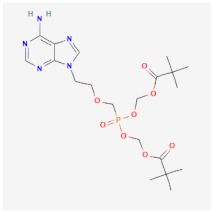	Useful for Lamivudine-resistant patients. It functions by the inhibition of HBV DNA polymerase.polymerase.	Lower efficiency when compared to newer drugs and increased resistance at prolonged usage, which needs strict monitoring. It also has nephrotoxic effects.	[45,46]
Emtricitabine (FTC)	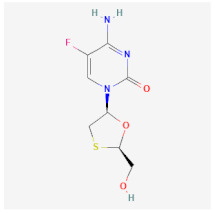	Functions as a combined therapy with tenofovir for co-infection (HIV-HBV) by incorporating viral DNA and preventing replication.	Reduced potency due to cross-resistance in combined therapy with Lamivudine.	[47,48]
Entecavir (ETV)	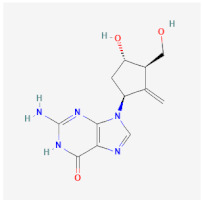	Effectively terminates HBV DNA polymerase chain, resulting in inhibition of HBV replication.	Ineffective for HIV-HBV co-infection and not recommended for HBV patients with Lamivudine resistance.	[49,50]
Tenofovir disoproxil fumarate (TDF)	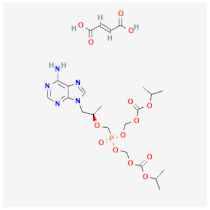	Incorporates into the viral DNA, resulting in the inhibition and premature termination of HBV DNA polymerase.	Prolonged usage results in renal and bone mineral problems. Caution and monitoring are also required for patients with associated kidney problems.	[51,52]
Tenofovir alafenamide (TAF)	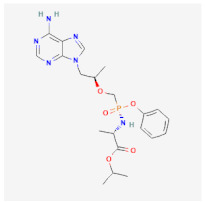	TAF is an improved brand of TDF. It inhibits HBV DNA polymerase replication.	TAF has kidney and bone problems, including other side effects in comparison to TDF.	[53,54]
Telbivudine (LdT)	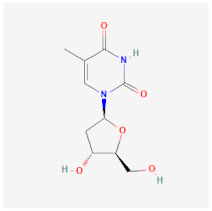	An analog of thymidine functions by inhibition of HBV DNA polymerase, leading to the decreasing of viral load andand DNA chain termination.	Ineffective as a monotherapy and not recommended for HIV-HBV co-infection patients.	[54,55]
Didanosine (ddl)	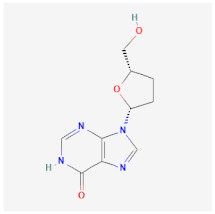	An analog of dideoxynucleoside. Functions by inhibition of viral DNA replication.	Limited usage due to adverse drug effects such as pancreatitis toxicity and peripheral neuropathy.	[56,57]
Zidovudine (AZT/ZDV)	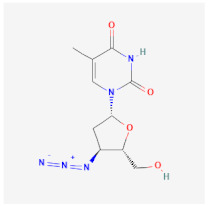	It causes premature termination of viral replication when it binds to the viral DNA.	Safer alternatives are preferred because they are highly toxic and suppress the bone marrow. It also causes anemia and gastrointestinal problems.	[58,59]
Stavudine (d4T)	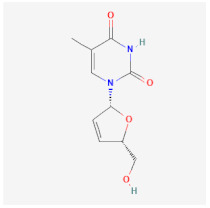	Functions by inhibiting reverse transcription and viral DNA replication.	It has limited use in treating HBV patients because of severe side effects such as lactic acidosis, peripheral neuropathy, and lipodystrophy.and	[60,61]
Lopinavir/Ritonavir (LPV/r)	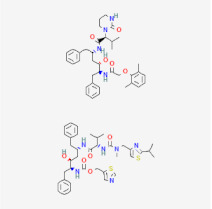	Function as a combinatory therapy for inhibition of HBV replication, by modulating the host immune response.	Although not originally approved as HBV drugs but have shown a level of potency in reducing the HBV DNA viral load. They have side effects, including gastrointestinal and lipid malfunctions.	[62,63,64]

**Table 2:** Comparative analysis of HIV and HBV replication mechanisms.

HIV	HBV	References
Binds to the CD4 or the C-C chemokine receptor type 5 (CCR5), a co-receptor on host immune cells. This facilitates the attachment to the host cell membrane, which allows the entrance of the viral RNA.	HBV gains entrance into the host using the hepatocyte sodium taurocholate co-transporting polypeptide (NTCP) receptor.	[65,66]
HIV employs reverse transcriptase for converting single-stranded RNA to double-stranded DNA immediately after it gains entrance into the host’s cells. This mechanism is essential because the virus integrates into the genome of the host, which allows it to take over the host’s replication processes.	HBV also employs a reverse transcription mechanism even as a DNA virus. The partially double-stranded relaxed circular DNA (rcDNA) is converted into covalently closed circular DNA (cccDNA) immediately after it gains entrance into the host’s cells. Subsequent reverse transcription of the pre-genomic RNA to form new viral DNA is enabled by the cccDNA.	[67,68]
The integration of HIV reverse-transcribed DNA into the host genome establishes a latent reservoir, which poses a serious setback in the HIV therapeutic process.	Naturally, there is no full integration of HBV into the genome of the host’s DNA, but the cccDNA of HBV stays inside the nucleus, acting as a precursor, which enables a steady viral transcription and replication. This partial integration of the cccDNA of HBV into the nucleus results in challenges in eradicating HBV.	[69,70]
HIV leverages its host’s cellular mechanisms for the transcription and translation of viral proteins. It uses the host polymerase to achieve the transcription and translation processes.	HBV also leverages its host’s cellular mechanisms for the transcription and translation of viral proteins. HBV also uses the host polymerase to achieve the transcription and translation processes.	[71,72]
HIV undergoes assembling and maturing after viral replication, which is discharged into the cell of the host (exocytosis). Protease (enzyme) is essential for HIV maturation.	HBV also undergoes assembling and maturing after viral replication, which is discharged into the cell of the host (exocytosis).	[73,74]

**Table 3:** Medicinal plant extracts and mode of action against Hepatitis B virus.

Type of Natural Product	Extract Type	Mode of Action	IC_50_ (mg/mL or μg/mL)	Reference
Scutellaria barbata D.Don	Ethanolic extract	Inhibition of HCC cellular proliferation (HepG2.2.15 and Hep3B cells after 72 h), suppressed levels of HBsAg and HBV-DNA, reduced colony size and number, reduced tumor volume and weight in mice after treatment	1.19 mg/mL	[89]
Oldenlandia diffusa (Willd.) Roxb	Ethanolic extract	A similar mode of action as Scutellaria barbata, inhibiting HCC cell proliferation and HBV-related markers	2.28 mg/mL	[89]
Scutellaria barbata D.Don and Oldenlandia diffusa (Willd.) Roxb	Ethanolic extract	Combined extract reduced tumor volume and colony size, and suppressed HBsAg and HBV-DNA	1.06 and 0.92 mg/mL	[6]
Cananga odorata	Methanolic extract	Inhibition of extracellular HBV DNA production (72.7% in Hep38.7-Tet cells), reduced HBsAg production in HepG2-NTCP cells (58% at 7–8 days post-infection)	56.5 μg/mL, 142.9 μg/mL	[90]
Guiera senegalensis	Dichloromethane extract	Inhibition of HBsAg expression (66%) in HepG2.2.15 2, comparison with Pulicaria crispa (55.3%) and Fumaria parviflora (54.7%)	10.65 μg/mL	[91]
Pulicaria crispa	Ethyl acetate extract	Inhibition of HBsAg production in HepG2.2.15 cells	14.45 μg/mL	[91]
Fumaria parviflora	Hexane extract	Inhibition of HBsAg production in HepG2.2.15 cells	35.44 μg/mL	[91]

## Data Availability

No new data were created or analyzed in this study.
